# *Enterococcus faecalis* bacteremia: please do the echo

**DOI:** 10.18632/aging.102619

**Published:** 2019-12-12

**Authors:** Anders Dahl, José M. Miro, Niels E. Bruun

**Affiliations:** 1 Department of Cardiology Herlev Gentofte University Hospital, Denmark; 2 Department of Cardiology Bispebjerg Hospital, Denmark; 3 Infectious Diseases Service, Hospital Clinic - IDIBAPS, University of Barcelona, Barcelona, Spain; 4 Department of Cardiology Zealand University Hospital, Roskilde; 5 Clinical Institute Copenhagen University, Copenhagen, Denmark; 6 Clinical Institute Aalborg University, Denmark

**Keywords:** endocarditis, bacteremia, health-care associated, enterococcus faecalis, echocardiography

Infective endocarditis (IE) caused by *Enterococcus faecalis* (*E. faecalis*) is a disease of the elderly with an increasing incidence, often health-care associated and with in-hospital mortality rates around 10-20% [1]. *E. faecalis* IE is notoriously challenging to diagnose due to unspecific symptoms, often presenting with a complex clinical picture with low-grade fever and only moderately elevated infectious parameters [1].

In a newly published prospective multicenter study using echocardiography to screen *E. faecalis* bacteremia patients, we found an IE prevalence as high as 26% [2]. The 344 included patients with *E. faecalis* bacteremia had a mean age of 74 (±12) years confirming that it is indeed a disease of the elderly. The key feature of the study was that echocardiography was performed in all patients including transesophageal echocardiography (TEE) in 74% [2]. Transthoracic echocardiography (TTE) missed vegetations in half of the cases where TEE demonstrated vegetations, underlining the importance of TEE [2].

An earlier retrospective study performed in the same catchment area included 647 patients with *E. faecalis* bacteremia and found an IE prevalence of 12% [3]. The higher prevalence in our recent study [2] is in part explained by the general increasing prevalence of *E. faecalis* IE. However, we believe that the substantially lower IE prevalence in the retrospective study is also related to missed cases of IE due to much lower examination rates with TTE in 36% and TEE in 18% of the bacteremia cases [3]. To further substantiate this, we have analyzed unpublished data from the retrospective study to evaluate the likelihood of missed IE cases due to insufficient examinations [3]. From the 78 patients with IE, ten were not diagnosed until relapse of *E. faecalis* bacteremia following insufficient primary work up (initially only three out of ten had TTE and none had TEE). In the patients not diagnosed with IE, 37 patients had relapse of *E. faecalis* bacteremia and 28 (75%) of these patients were insufficiently examined with either no echocardiography at all (15), only initial echocardiography and not at relapse (seven) or only TTE at relapse (six) [3]. From analyzing the patient charts, the impression was that the primary reason why these patients were insufficiently examined, was that they were widely spread in different surgical and general internal medicine departments and not referred to an IE specialist nor for cardiac imaging. The combined evidence from these two studies illustrates why it is crucial to perform both TTE and TEE in *E. faecalis* bacteremia and stress the importance of disseminating this knowledge beyond the narrow spectrum of IE specialists [2,3].

When it comes to diagnosing *E. faecalis* IE, international guidelines only include community acquired enterococcal bacteremia in the absence of a primary focus as a major criterion of a typical microorganism consistent with IE [4]. In our opinion, this definition is outdated since health-care associated IE is increasingly important due to the growing population of elderly and comorbid patients with many contacts to the health-care system [1]. Despite the fact, that community-acquired bacteremia is consistently associated to *E. faecalis* IE, our new data confirm earlier findings that around half of the *E. faecalis* IE cases (52%) are health-care associated [2]. In that perspective, we suggest that the ESC diagnostic criteria are updated to include health-care associated *E. faecalis* bacteremia to better reflect contemporary data.

In the clinical setting, while planning the work up in older co-morbid patients with *E. faecalis* bacteremia, it is possible to encounter the argument that TEE seems futile since the patient likely will not qualify for cardiac surgery. However, these patients can still benefit from being diagnosed with *E. faecalis* IE and thereby receiving extended antibiotic treatment. This apprehension is supported by a study describing several older, not operated patients with relapse of *E. faecalis* IE, handled by extended antibiotic treatment successfully continued into lifelong suppressive antibiotic treatment [5]. Combined with the new evidence showing an IE prevalence of up to 26% in *E. faecalis* bacteremia, we propose that TTE/TEE is performed in patients with *E. faecalis* bacteremia unless there is a specific reason not to do so [2]. A reason to withhold echocardiography could be patients presenting with none of the six risk factors observed in the prospective study (prosthetic heart valve, community acquisition, ≥3 positive blood culture bottles, unknown portal of entry, monomicrobial bacteremia or immunosuppression) [2], and without other known high risk conditions such as previous IE, cardiac devices or renal failure treated with dialysis.

In addition, patients diagnosed with *E. faecalis* IE and unknown primary focus of infection have an increased risk of having colorectal neoplasms including colorectal cancer and therefore colonoscopy is highly recommended in these patients [6].

When attempting to predict the future development in *E. faecalis* IE it is relevant to consider two important studies. Firstly, a newly published nationwide population based study showed that *E. faecalis* bacteremia had the highest IE prevalence (17%) among typical IE bacteria [7]. The prevalence of *E. faecalis* IE increased significantly across the period 2010-2017 and with increasing patient age [7]. Secondly, an international multicenter study found enterococci to be the most common causative agent of IE in patients with Transcatheter Aortic Valve Implantation [8]. Combining this knowledge with the development in demographics and health care systems comprising older and older patients with more and more implanted prosthetic material in the heart, *E. faecalis* IE is a disease that is bound to further increase in prevalence and importance.

In summary, we recommend adjusting IE guidelines to encompass health-care associated bacteremia as a diagnostic criterion in *E. faecalis* IE, to increase the general focus on *E. faecalis* bacteremia and as part of the work up; “please do the echo”.
